# Finding harmony so the music plays on: pragmatic trial design considerations to promote organizational sustainment of an empirically-supported behavior therapy

**DOI:** 10.1186/s13722-016-0049-6

**Published:** 2016-01-22

**Authors:** Bryan Hartzler, K. Michelle Peavy, T. Ron Jackson, Molly Carney

**Affiliations:** Alcohol and Drug Abuse Institute, University of Washington, Box 354805, 1107 NE 45th Street, Suite 120, Seattle, WA 98105-4631 USA

**Keywords:** Pragmatic trial design, Contingency management, Therapy sustainment

## Abstract

**Background:**

Pragmatic trials of empirically-supported behavior therapies may inform clinical and policy decisions concerning therapy sustainment. This retrospective trial design paper describes and discusses pragmatic features of a hybrid type III implementation/effectiveness trial of a contingency management (CM) intervention at an opioid treatment program. Prior reporting (Hartzler et al., J Subst Abuse Treat 46:429–438, 2014; Hartzler, Subst Abuse Treat Prev Policy 10:30, 2015) notes success in recruiting program staff for voluntary participation, durable impacts of CM training on staff-level outcomes, provisional setting implementation of the intervention, documentation of clinical effectiveness, and post-trial sustainment of CM.

**Methods/design:**

Six pragmatic design features, and both scientific and practical bases for their inclusion in the trial, are presented: (1) a collaborative intervention design process, (2) voluntary recruitment of program staff for therapy training and implementation, (3) serial training outcome assessments, with quasi-experimental staff randomization to either single or multiple baseline assessment conditions, (4) designation of a 90-day period immediately after training in which the setting implemented the intervention on a provisional basis, (5) inclusive patient eligibility for receipt of the CM intervention, and (6) designation of two staff as local implementation leaders to oversee clinical/administrative issues in provisional implementation.

**Discussion:**

Each pragmatic trial design feature is argued to have contributed to sustainment of CM. Contributions implicate the building of setting proprietorship for the CM intervention, culling of internal staff expertise in its delivery, iterative use of assessment methods that limited setting burden, documentation of setting-specific clinical effectiveness, expanded penetration of CM among staff during provisional implementation, and promotion of setting self-reliance in the oversight of sustainable implementation procedures. It is hoped this discussion offers ideas for how to impact local clinical and policy decisions via effective behavior therapy dissemination.

## Background

Decades ago, the National Institute on Health [1] identified clinical trials as “the most definitive tool for evaluation of the applicability of clinical research” with precision in controlled therapy comparisons expected to improve the quality and cost-effectiveness of health services. This hope remains despite persistent debate among researchers, policy makers, and the treatment community as to where on an internal-external validity pendulum most useful trial designs lie. Many traditionalists tout the randomized controlled trial (RCT) as a gold standard methodology [2]. Benefits ascribed to RCTs, as outlined by Friedman et al. [3], are that they: (1) eliminate bias in patient assignment to treatments, (2) produce, in theory, comparable groups to minimize potential 3rd-variable influences, and (3) assure the validity of corresponding statistical tests. Counterarguments focus on the poor representativeness of recruited patients and study settings in RCTs, as well as real-world inapplicability of many procedures and outcomes [4]. Debates about trial design are further perpetuated by lack of consensus among systematic reviews of evidence generated by RCTs versus time-series and case-controlled designs [5–9].

An extension of this debate contrasts explanatory and pragmatic trials, dating back half a century to Schwartz and Lellouch’s [10] characterization of the former as testing a therapy’s causal relations to its outcomes and the latter as addressing its implications for health services policy. Perceived overreliance on explanatory trials has prompted outcries for research with greater real-world applicability [11, 12]. Flay [13] was an early advocate that in order to address health service policy implications, trials must focus beyond patient outcomes to broader issues of therapy implementation and sustainment. This notion now appears prophetic, given subsequent Institute of Medicine [14] reporting of ‘research-to-practice gaps’ in addiction care. Frequent and continual citation of this report among addiction treatment researchers nearly two decades later underscores that there is still much to learn about the implementation and sustainment of empirically-supported behavior therapies in addiction care settings.

In the current funding climate, advancing the science of behavior therapies necessarily involves efficient evaluation methods. Hybrid designs that blend traditional features of efficacy and effectiveness trials are a suggested means of expediting knowledge about therapy impacts [15–17]. In a similar vein, Curran et al. [18] propose a typology of hybrid trial designs conjointly addressing therapy effectiveness and implementation. This includes: (1) *hybrid type I trials*, that principally determine therapy effectiveness and secondarily explore setting factors influencing its implementation, (2) *hybrid type II trials*, with co-primary aims to test therapy effectiveness and utility of implementation strategies, and (3) *hybrid type III trials*, which examine implementation strategies for an already empirically-validated therapy and secondarily evaluate resulting clinical effectiveness. Just as efficacy/effectiveness hybrid designs efficiently test a therapy’s clinical impacts [17], so to should implementation/effectiveness hybrid designs [18] spur expeditious insights into its health service policy implications.

Choices faced by behavior therapy researchers about trial design will be influenced by a therapy’s existing empirical support. With respect to trials conducted in addiction treatment settings, contingency management (CM) is comprehensively studied as a therapy wherein behavioral reinforcement principles shape patient treatment adherence. Already a focus of 200+ published trials in such settings, meta-analyses of CM note reliable therapeutic effects [19–21]. Further, clinical effectiveness is demonstrated in paired NIDA Clinical Trials Network studies [22, 23]. Nevertheless, optimism about CM dissemination is tempered by low rates of treatment community adoption [24–26], with identified sources of reluctance that encompass fiscal, logistical, and ideological barriers [27, 28]. Collectively, this positions CM well for the conduct of Curran et al.’s [18] hybrid type III trials—wherein primary units of analysis for trial outcomes consist of treatment sites (i.e., therapy implementation costs, logistical feasibility, sustainability) and their existing staff members (i.e., therapy skill, knowledge, attitudes, eventual adoption).

In a CM-focused hybrid type III trial at an opioid treatment program (OTP), Hartzler et al. [29] tested as implementation strategies a collaborative intervention design process, active learning strategies to cull staff delivery skills and adoption readiness in CM training, and designation of two OTP staff as local implementation leaders. As previously reported [29, 30], trial findings include: (1) effective recruitment of 80+ % of OTP staff for voluntary participation, (2) robust, durable training impacts on CM delivery skill (*d* = 2.43) and adoption readiness (*d* = .88), 3) 100 % penetration among CM-trained staff during a 90-day period in which implementation by the setting was conducted on a provisional basis, (4) medium effects on targeted patient behaviors (*d* = .46–.53, relative to historical control patients), (5) qualitative impressions of CM affordability and compatibility among OTP management at trial conclusion, and (6) post-trial setting report of continuous two-year sustainment of CM among routine service provisions. Collective outcomes suggest that pragmatic design features of this single-site trial may offer useful ideas for how to impact local clinical and policy decisions via effective behavior therapy dissemination.

Johnson et al. [31] offer a musical analogy for the challenges of pragmatic trial design, likening the rigorous structure of traditional treatment research to classical music and the fluidity of clinical practice to improvisational jazz. To extend the analogy, a well-conceived pragmatic trial will include design features that harmonize these musical styles so findings prompt data-informed, sustainable services. This paper—authored by a university-based investigator (BH), the OTP director (TRJ), an implementation leader (KMP), and a managerial staff member (MC) in the aforementioned CM trial—takes a retrospective view in describing six pragmatic design features outlined in Table 1. The lead author (in his capacity as a behavior therapy dissemination researcher) embraced a role of therapy purveyor in seeking to disseminate CM to this community setting, insofar as he conceived the trial design, provided organizational consultation and training of staff, evaluated clinical impacts of therapy implementation, and assessed OTP decisions about its eventual sustainment. Herein, these trial design features are detailed, followed by discussion of underlying scientific and practical rationales for their inclusion in the trial as well as perceived influence on trial outcomes and eventual sustainment of the CM intervention in this OTP setting.

**Table 1:** Six pragmatic trial design features intended to foster intervention sustainment at the participating opioid treatment program

Feature	Feature	Intended benefit
1. Collaborative intervention design process involving both the study investigator (a CM purveyor) and the OTP director	Shared design responsibility allowed intervention to be theoretically-informed and matched to setting-specific fiscal and logistical implementation capacities	Enhanced ‘ownership’ of intervention by setting management and staff; increased likelihood of post-trial sustainment
2. Voluntary recruitment of OTP staff for therapy training and subsequent delivery to patients on a provisional basis	‘Real-world’ conditions would require that this CM intervention be delivered by direct-care staff amidst their regular contact with patients in usual care	Culling of internal expertise in the setting; greater opportunity to identify barriers and contextual adaptations for the intervention
3. Serial training outcome assessments, with quasi-experimental staff randomization to single versus multiple baseline conditions	Longitudinal assessment of staff-based outcomes (i.e., skills, knowledge, adoption readiness) was needed, but with means to account for assessment reactivity	Sufficient staff data collection absent undue setting burden; avoidance of contamination concerns given training of intact staff group
4. Provisional 90-day implementation period for CM-trained staff to deliver the focal intervention to their caseload patients	An interim period during which setting staff would form experience-based impressions of intervention feasibility as well as determine site-specific clinical effectiveness	Setting able to make proximal, and informed decisions about the feasibility, effectiveness, and sustainability of the intervention
5. Broadly inclusive patient eligibility during provisional implementation, comparison to matched group of historical control patients	Heterogeneity of OTP setting patients suggested need to maximize generalizability in documenting the feasibility	Meaningful conclusions about site-specific intervention utility; more expedient accrual and effectiveness of the intervention of staff implementation experiences
6. Designation of local implementation leaders, included in consultative planning discussions with purveyor and OTP director	The OTP was well-positioned for autonomous period of provisional implementation, and this was expected to better inform setting decision about therapy sustainment	Internal support for implementation efforts integrated into *supervision*-*as*-*usual* instead of a resource-intensive fidelity rating system

Pragmatic design features associated with single-site trial conducted by Hartzler et al. [29] testing implementation of a contingency management (CM) intervention at a community-based opioid treatment program (OTP)

## Methods/design

### Ethics, consent, and permissions

This implementation/effectiveness hybrid type III trial was conducted with full approval of the University of Washington Institutional Review Board. Participating OTP staff members provided informed consent in writing prior to their voluntary involvement in any trial activities.

### Pragmatic trial design feature #1: Collaborative intervention design

The intent was to create a clinically-useful intervention matched to OTP implementation capacity (i.e., operating budget, staffing resources), for which community treatment perspectives have proven salient in past CM dissemination [32, 33]. Initially, the purveyor oriented the OTP director to core CM tenets [34]; specification of an observable target behavior, timely provision of tangible reinforcers upon its observance, and withholding of reinforcement in its absence. Informed by contextual insights into setting needs and resources, the OTP director specified: (1) patient population (i.e., new enrollees), (2) target behavior (i.e., attendance of weekly counseling visits), (3) reinforcers to be provided (i.e., $5 gift cards to local vendors, take-home doses), and (4) reinforcement system (i.e., a point-based token economy). The hope was to enhance engagement of the setting’s 35–40 new enrollees each month, increase their interaction with staff, incentivize counseling attendance via affordable reinforcers, and create manageable procedures for existing staff to implement. The purveyor devised a reinforcement schedule—the rate at which points were to be earned, and thresholds at which patients could exchange earned points for available reinforcers—incorporating operant conditioning principles (i.e., priming, escalation/reset) to enrich clinical impact. The design process concluded with conjoint review of the intervention by purveyor and OTP director, and formal setting approval for its provisional implementation.

### Pragmatic trial design feature #2: Voluntary staff participation

Substantive role of the OTP director in intervention design ensured its consideration of therapy-relevant staff attributes like their interest, available time, and professional capability. Accordingly, the intervention capitalized on regularly scheduled contact with new patients, direct exposure to therapeutic benefits experienced by patients, and concrete procedures intended to promote reliably skillful staff delivery. Documentation of CM-related data at each patient visit (i.e., running point total, reinforcers received) in electronic medical records offered ongoing means of case-specific fidelity monitoring. Recognizing professional autonomy as a staff value, the OTP director afforded individual staff freedom to choose if they would participate in the trial, and to what extent they attended training and then implemented CM on a provisional basis over a predetermined 90-day period. As previously-reported [29], 80+ % of staff consented to voluntarily participate and all CM-trained staff who had opportunity to implement the intervention did so.

### Pragmatic trial design feature #3: Serial training outcome assessments

Broader therapy training literature documents clinician variability in acquisition and maintenance of therapy-relevant skills, knowledge, and attitudes [35, 36]. Thus, longitudinal assessment of a sufficient staff sample was needed, with means to account for assessment reactivity (i.e., ‘practice effects’). Accordingly, serial training outcome assessments—prior to, after, and three months following training—were conducted with quasi-experimental staff randomization to single vs. multiple baseline assessment conditions. The primary index in all assessments was independently-rated behavioral fidelity in a standardized patient (SP) interaction, which minimized setting burden by circumventing the selection biases and personal intrusion inherent in observation/recording of patient sessions. As consistent and consequence-free clinical stimuli, SPs are a validated means to produce reliable fidelity estimates for behavior therapy implementation in addiction settings [37]. In each assessment, the SP interaction was supplemented by an applied CM knowledge instrument [38], multiple-choice test of CM principles [39], and adoption readiness scale [28]. Efforts to minimize setting burden succeeded insofar as all assessments were completed. Resulting data sufficiently demonstrated robust longitudinal training impacts and absence of assessment reactivity [29].

### Pragmatic trial design feature #4: A post-training period of provisional implementation

The purveyor and OTP director agreed in advance to a 90-day period following staff training during which CM-trained staff members had opportunity to implement the intervention on a provisional basis with new patients assigned to their caseloads. Staff participation in the structured CM training was the principal preparatory effort, though augmented by a set of four consultative planning meetings of the purveyor and five managerial staff for 30 min prior to each staff training sessions. Meetings focused on preparatory implementation activities (i.e., reinforcer purchasing/accounting, electronic medical record system modification), and provided the purveyor informal means of formative evaluation of setting readiness. These preparatory efforts were concurrent to the staff training process and similarly predated a 90-day provisional implementation period, after which setting management had the option to sustain, amend, or discontinue use of the intervention. The specific date of onset for this provisional period of implementation was left to the OTP director’s discretion (ultimately initiated 2 weeks after staff training). Summative evaluation of implementation experiences occurred at the conclusion of this 90-day period, in the context of a group interview with managerial OTP staff wherein their qualitative impressions of affordability, compatibility, and sustainability were elicited [30].

### Pragmatic trial design feature #5: Broad patient eligibility

The trial sought broad patient eligibility during provisional therapy implementation, with three issues challenging this effort. The 1st was intervention’s specific targeting of new patients, which precluded eligibility of existing patients. Second, implementation was only possible with patients assigned to the caseload of a staff member trained to deliver the intervention. Though most of the OTP staff participated in CM training, this left a minority whose new patients were ineligible. Finally, as the OTP’s patient enrollment included admission of a subset of individuals on 180-day opioid detoxification, the CM intervention was slightly adapted to remove take-home medication doses from among available reinforcers for these persons. Despite these challenges, the CM intervention reached 106 OTP patients during the 90-day period—exceeding that which was suggested for comparison to historical control patients to detect a meta-analytic mean effect size (*d* = .42) of CM efficacy trials [20]. A further encouraging sign for the OTP setting was its emergence from the 90-day period of provisional implementation with report of no persisting or unresolved problems of patient eligibility for receipt of the CM intervention.

### Pragmatic trial design feature #6: Designation of staff as local implementation leaders

Provisional therapy implementation was governed autonomously by the OTP for several reasons. First, the intervention had clear support of setting leadership given its design process. Second, staff training included iterative rehearsal of intervention delivery and performance-based feedback, both suggested methods predictive of CM fidelity [40]. Third, all SP interactions were scored with a fidelity instrument [41] on which all trained staff members exceeded a competency benchmark [42]. With the setting poised for provisional implementation without substantial purveyor involvement, the OTP director designated two program staff to be local implementation leaders—with responsibilities divided into clinical (i.e., staff supervision) and administrative (i.e., reinforcer purchasing/accounting) tasks. Both had participated in all consultative planning meetings, and were well-positioned to address issues arising in provisional implementation. Nevertheless, channels of passive purveyor support were put in place. One was continual purveyor availability for phone/email consultation, utilized sparingly with a handful of staff-initiated contacts over 90 days. The other was creation of an on-site ‘CM training library,’ with master copies of all training materials kept in a designated location for convenient staff access. The 90-day period of provisional implementation then proceeded at the OTP with oversight of CM-trained staff integrated into the setting’s *supervision*-*as*-*usual* practices (i.e., semi-weekly individual case review, weekly staff meetings).

## Discussion

Six pragmatic features of the focal hybrid type III implementation/effectiveness trial have been detailed. Scientific and practical bases underlying the inclusion of each feature will now be discussed, along with their perceived contribution to post-trial CM sustainment in the setting.

### Collaborative intervention design

In many explanatory trials, the purveyor has a clear conceptualization of the focal therapy from the outset that precludes its contextualization to the clinical setting. Pragmatic trials are apt to approach this differently, as local clinical and policy decisions need to account for between-setting variance in organizational attributes like staffing resources, service structure, and patient census characteristics [11]. Among behavior therapies for addiction settings, CM is noted for its capacity for contextual adaptation [43]. The conceptual clarity of core CM tenets [34] aids this, leaving a set of malleable features (e.g., eligible patients, target behavior, available reinforcers, reinforcement system) to then be flexibly defined according to setting needs and resources.

To what extent did the intervention design process contribute to sustainment of CM? A great deal, we believe. The purveyor could have instead simply advocated that the OTP replicate procedures of Higgins et al.’s [44] escalating voucher or Petry’s [45] prize-based ‘fishbowl’ methods that demonstrated prior efficacy. However, the OTP director regarded both methods a mismatch for the setting’s limited fiscal resources, large patient census, and idiographic structure of patient services. A common treatment community sentiment is that new practices are adopted only if “they don’t conflict with treatments already in place” [46]. In addition to matching its fiscal resources, the collaborative design process produced an intervention that was logistically compatibility with existing services—as staff monitored the target behavior, tracked points, and delivered earned reinforcers amidst usual care in weekly counseling visits. Finally, OTP director specification of malleable intervention features begot a sense of ownership that otherwise may not have developed, prompting emergence of intervention proprietorship among setting staff that then guided generally positive provisional implementation experiences. Thereafter, the OTP director recreated the spirit of the collaborative design process by inviting staff feedback about potential amendments to malleable intervention features, which amplified enthusiasm and commitment among setting staff, prior to its formal inclusion in the setting’s treatment manual.

### Voluntary staff participation

In many explanatory trials, research therapists are hired externally—sought by virtue of an affinity for, allegiance to, and experience with a focal therapy. Such therapist selection serves well the aims of explanatory trials, as do common practices of closely-supervised practice cases, therapy implementation apart from routine clinic services, and expert scrutiny via fidelity review of patient sessions. A chief criticism of such procedures for therapist selection, training, and implementation is limited external validity with respect to staffing expertise, time, and resources in community settings [4]. Related concerns center on typically busier, eclectic clinical practice routines of community health providers, and ambivalence commonly held toward adoption of unfamiliar behavior therapies [28, 47–49]. The stance in this trial—that setting staff voluntarily participate in therapy training and implementation—is consistent with published interdisciplinary perspectives that pragmatic trials be designed to offer professional development opportunities for staff to hone therapeutic skills via direct participation in quality improvement efforts [50–52].

To what extent did voluntary staff participation in therapy training and implementation contribute to eventual CM sustainment? In our eyes, the contribution was substantive. Many CM trials, including those attributed with demonstrating community effectiveness via NIDA’s Clinical Trials Network [22, 23], rely entirely on external staffing. This translates poorly to the realities that community treatment program directors face, and helps explain a 12 % rate of post-trial CM sustainment among CTN programs [24]. Community treatment programs must rely on existing staff members, who are likely to vary greatly in interest and capability to adopt new therapies—and among whom mandated training may provoke negative reactance. Accordingly, trial recruitment of staff was governed by optional innovation decisions [53], with individual OTP staff entrusted to self-determine a participation level. An eventual product of this voluntary staff involvement in CM activities was the development of internal CM expertise in the setting, on which later decisions favoring sustainment of the therapy would rest. This is consistent with evidence from prior research wherein provisional experience with direct CM delivery predicted supportive attitudes toward eventual community-based implementation [54, 55]. Coupled with strong managerial support for the intervention, provisional implementation experiences among CM-trained staff prompted many to advocate that nonparticipating staff later undergo training so that the CM intervention would have broader reach within the OTP patient census.

### Serial training outcome assessments

In most explanatory trials, there is no report of longitudinal impacts of therapy training, perhaps due to investigator expectation that procedural fidelity is assured by selection of already capable, allegiant research therapists. Measurement is typically limited to initial verification of skillful delivery in supervised practice cases, and independent fidelity rating of therapy sessions subsequently conducted during the trial. These research therapists are free from many of the complexities and competing demands inherent in clinical practice [56], instead proceeding under fairly idyllic conditions with singular focus on closely adhering to manualized procedures for the identified therapy as delivered to select patients for whom it is thought particularly salient. This neglects salient dilemmas facing community treatment programs about how to develop and maintain internal expertise among its clinical staff. Such dilemmas encompass contributing clinician-level issues like time and philosophical congruence [57] and patient-level challenges such as prevalence of polysubstance use disorders and comorbid health conditions [58].

To what extent did serial training outcome assessments contribute to sustainment of CM? We believe scientific and clinical needs were effectively balanced, modeling suggested features of pragmatic trial measurement [59]: sufficient data collection, low setting burden, local clinical applicability, and opportunity to show sensitivity to change. As reported [29], quasi-experimental staff randomization to single versus multiple baseline assessment conditions documented nominal assessment reactivity across behavioral, intellectual, and attitudinal training outcomes. Notably, it did so without logistical challenges and contamination concerns inherent in experimental trial designs involving staff randomization to active training versus waitlist/control conditions. Resulting opportunity to train interested OTP staff as an intact group—and assess individual and collective training impacts on CM delivery skill, knowledge, and adoption readiness—was critical to assure adequate staff preparation for implementation. A key aspect was post-training documentation of all CM-trained staff exceeding a competency benchmark for delivery skill [42]. Absence of unresolved problems in provisional implementation, paired with trial documentation of durable training gains over 90 days, heightened setting confidence for prospects of post-trial sustainment.

### A post-training period of provisional implementation

In most explanatory trials, assessment of therapy implementation serves strictly scientific purposes. Available data are limited to fidelity ratings of therapy sessions by externally-hired, trained, and supervised research therapists delivering a focal therapy and/or comparative therapy approach. Statistical documentation that such ratings evidence treatment integrity (i.e., delivery as purveyor intended) and discriminability (i.e., delivery distinct from its comparator) are key scientific aims [60], and this understandably encompasses evaluation of therapy implementation in many trials. An unfortunate consequence is that issues complicating real-world therapy implementation are left unaddressed. Broadly, these relate to implementation costs (i.e., staff time required for therapy training and implementation, clinical supervision, necessary therapy materials or technology), contextual compatibility with setting structure (i.e., other clinical services, records systems), and observed penetration or reach (i.e., rates of staff adoption and/or patient exposure) in a setting [31, 61]. Absent serious consideration of such issues, sustainment of an empirically-supported behavior therapy for any meaningful period is difficult to imagine.

Did inclusion of an initial period during which CM-trained setting staff implemented the CM intervention with their patients on a provisional basis contribute to its eventual sustainment? Our answer is yes, eventually. Onset of this 90-day period shortly after staff training allowed provisional implementation experiences to occur as staff training gains were fresh. The 90-day duration was informed by published recommendation of conservative sampling and analytic methods, given greater patient heterogeneity encountered in pragmatic trials [62]. This length of time provided sufficient staff and patient exposure to CM, which informed discussion of possible intervention amendments amongst a designated committee of OTP staff. Setting decision about sustainment was initially deferred until results of a chart-based comparison of CM-exposed versus historical control patients were known. As had been true throughout the trial, purveyor citation of reliable therapeutic effects observed in extant CM literature conducted in addiction treatment settings was met with a familiar refrain that “none of those studies were conducted *here*” [63]. Upon later receipt of documentation of site-specific clinical effectiveness (*d* = .45–.53, [29]), the setting formally committed to sustain the CM intervention among routine service provisions, and required exposure of all untrained and prospectively-hired staff to the CM training curriculum.

### Broad patient eligibility

Explanatory trials use inclusion/exclusion criteria to select persons from a larger patient population for whom a focal therapy is thought particularly relevant. In CM trials in addiction settings, this often restricts recruitment to those with diagnosis or recent evidence of a single substance of abuse and absent medical or psychiatric comorbidity. This has clear advantages, as titration of treatment-seeking populations to those uniformly presenting with compartmentalized therapeutic needs simplifies both formulation and confirmation of hypothesized therapy effects. Of course, this significantly limits generalizability of resulting findings, as polysubstance use and multivariate health challenges are commonplace among enrollees at addiction settings [64, 65]. Selective therapy application, particularly when offering tangible rewards as contemporary CM approaches do, may spur reticence from staff and patients about issues of social justice. The more selectively applicable a therapy is framed to be, the lesser opportunity community settings and their staff have to witness relative advantages, compatibility, simplicity, trialability, and observability—all hallmark attributes of innovative practices that are widely adopted [53].

What impact did broad application of the intervention among OTP enrollees have on CM sustainment? During the 90-day period of immediate implementation on a provisional basis, broad patient eligibility facilitated timely accrual of a sufficient sample of CM-exposed patients to establish site-specific clinical effectiveness and 100 % penetration among CM-trained staff. Thus, qualitative impressions of its affordability and compatibility formally voiced by managerial staff at trial conclusion reflected direct experiences of most staff. With respect to the eventual sustainment of CM implementation at the OPT, broad application of the intervention among OTP enrollees was thought to strengthen perceived relevance of supporting evidence for setting-specific clinical effectiveness. An additional factor cementing the post-trial setting decision for CM sustainment was the vocal positive feedback about the intervention that staff reported receiving from their CM-exposed patients [29]. Broad patient eligibility enabled a greater proportion of new OTP enrollees to serve as sources of this informal feedback.

### Designation of staff as local implementation leaders

In explanatory trials, therapy implementation is closely monitored to ensure what occurs is as the purveyor intends. This is understandable, given a principal aim of such trials to confirm a therapy’s hypothesized effects. Pragmatic trials, in contrast, seek to balance competing needs of a clinical setting: (1) availability of purveyor support in initial therapy implementation, and (2) organizational autonomy so that internal expertise is culled to support independent sustainment. As Johnson et al. [31] suggest, implementation is facilitated by therapy integration into the flow of existing clinical practices. Thus, it is critical that a purveyor be available to support such efforts but without unnecessary involvement in their conduct. This is consistent with phased models of therapy implementation [66, 67], wherein removal of purveyor support and self-governed therapy sustainment by the clinical setting are intended endpoints.

Did designation of two OTP staff as local implementation leaders spur CM sustainment? One local implementation leader oversaw staff via supervision-as-usual practices at the OTP for which (commensurate with resources available in many clinical settings) time-intensive use of observational fidelity systems is impractical. This was sufficient to prevent the deterioration of initial training gains often observed after therapy training [68, 69], and to document therapeutic impacts similar to those of trials where community staff received active purveyor supervision [70–72]. The 2nd local implementation leader coordinated administrative procedures, for which the OTP emerged from provisional implementation absent unresolved issues. These collective actions contributed to setting impressions of intervention affordability and compatibility. Had local implementation leader voiced strong concern, evidenced inability to keep up with duties, or otherwise demonstrated need for active purveyor support during provisional implementation, setting enthusiasm for CM sustainment would surely have attenuated. Instead, provisional implementation experiences maintained, if not strengthened, enthusiasm for CM in the setting. Notably, supervision-as-usual practices persisted after the trial, with previously-untrained staff later exposed to the CM training curricula. Administrative procedures were largely maintained post-trial, with effort to improve efficiency of tracking systems and manage interdepartmental coordination amidst a two-year period of organizational growth. Further, local implementation leaders continued to serve as repositories for CM-relevant feedback from staff and patients.

## Conclusions

In this retrospective trial design paper, the contribution of pragmatic design features of a hybrid type III implementation/effectiveness trial to the eventual two-year sustainment of a CM intervention is described. Pragmatic design considerations had implications for how: (1) the intervention was designed, (2) OTP staff were recruited for trial involvement, (3) impacts of training on staff implementation outcomes were documented, (4) provisional implementation and resulting clinical effectiveness were assessed, (5) broad eligibility among a patient population occurred, and (6) localized staff leadership was culled to support setting autonomy in establishing sustainable implementation procedures. Though the single-site nature of this trial may obscure complexities encountered in interagency therapy dissemination [73], it is hoped this description and discussion of pragmatic design features may spark ideas for future translational work concerning empirically-supported behavior therapies in community addiction settings.

Additional systemic processes enacted by the OTP in its two-year CM sustainment bear mentioning. One was iterative gathering of CM-related feedback from stakeholders, including clinical staff across service lines and (when possible) patients. As a result, creative uses of positive reinforcement permeated staff discussions and prompted later creation of other CM programming in this and two other newly-opened clinics governed by the treatment organization. Likewise, input was elicited from administrative staff to refine tracking systems for fiscal aspects of all CM programming. A 2nd systemic process involved staffing re-organization, with resources dedicated to create a ‘CM specialist’ position to coordinate sustainment of the focal intervention and introduction of other CM programming across OTP service lines. A 3rd systemic process involved a philosophical shift away from voluntary staff involvement in training/implementation activities [noted as examples of Rogers’ [53] optional innovation-decisions during the trial]. A necessary post-trial shift to authority innovation-decisions (i.e., system-wide adoption determined by those in authority) [53] mirrored that predictive of expedient change in complex systems like healthcare organizations. To prevent potential circumvention by uninterested staff members, internal communication from setting leadership highlighted the voluminous empirical support for CM and setting-specific evidence of the focal intervention’s clinical utility. Collectively, post-trial systemic processes enabled the OTP to apply CM principles to a breadth of setting goals and patient needs, and it remains poised to effectively respond to future challenges as they emerge.

To conclude, we hope this discussion reinforces the earlier sentiments of Schwartz and Lellouch [10], Flay [13], Rothwell [4], and others about the value of designing trials with sufficient attention to external validity. In looking back to this single-site trial to dissect its design features and the decisions underlying their inclusion, the intent is not to offer up a specific blueprint for other trialists to follow. Rather, it is hoped this work may prompt thoughtful and collaborative discussion about the design and conduct of future behavior therapy trials amongst therapy purveyors and community treatment settings with whom they partner. Such discussions enhance the likelihood of mutually-beneficial endeavors, for which resulting findings can then substantively guide local clinical and policy decisions about health services the public receives. In this example, a hybrid type III implementation/effectiveness trial predated sustainment of an empirically-supported CM intervention by an OTP. This is a reflection of efforts to harmonize rigor in scientific aims with appreciation for the fluidity and practicalities inherent in clinical practice. Insofar as CM remains firmly embedded in this addiction treatment organization’s routine service provisions, those collective efforts contributed to music that continues to play on.

## Abbreviations

RCT: randomized controlled trial
CM: contingency management
NIDA: National Institute on Drug Abuse
OTP: opioid treatment program
